# An extremely rare case of thymic squamous cell carcinoma complicated with B3 thymoma and myasthenia gravis: a case report

**DOI:** 10.1186/s40792-024-02025-2

**Published:** 2024-09-26

**Authors:** Maiko Atari, Hideki Kawai, Takuo Tokairin

**Affiliations:** 1 Department of Thoracic Surgery, Japanese Red Cross Akita Hospital, 222-1 Nawashirosawa, Saruta Kamikitate, Akita, 010-1495 Japan; 2 Department of Pathology, Japanese Red Cross Akita Hospital, 222-1 Nawashirosawa, Saruta Kamikitate, Akita, 010-1495 Japan

**Keywords:** Combined thymic carcinoma, Myasthenia gravis

## Abstract

**Background:**

Thymomas complicated with myasthenia gravis are conventionally treated during thoracic surgery. Particularly, invasive thymomas are resected alongside the surrounding organs. Here, we present a case where surgical and perioperative management was performed under the presumption of thymoma with myasthenia gravis. However, definitive pathology revealed the co-occurrence of B3 thymoma and thymic squamous cell carcinoma. This case highlights the unique presentation and exceptional rarity of thymomas that are complicated by myasthenia gravis and thymic carcinoma.

**Case presentation:**

A 65-year-old female presented with eyelid ptosis at our hospital. Following a comprehensive examination, the patient was diagnosed with myasthenia gravis. Her computed tomography (CT) scan revealed an anterior mediastinal tumor suggestive of a thymoma, prompting a referral to the Department of Thoracic Surgery. Moreover, preoperative assessment could not definitively exclude pericardial invasion. She subsequently underwent an extended thymectomy via a longitudinal sternal incision. The tumor exhibited partial invasion of the pericardium, necessitating resection and reconstruction. Definitive pathological examination confirmed the co-occurrence of B3 thymoma and thymic squamous cell carcinoma. Positive lymph node metastasis classified the patient as stage IVa according to the Union for International Cancer Control (UICC) TNM Classification of Malignant Tumors, 8th Edition, and she was started on adjuvant radiotherapy postoperatively. Currently, the patient remains under observation, with follow-up CT scans showing no signs of recurrence.

**Conclusions:**

This report describes an extremely rare case of thymoma complicated with myasthenia gravis and thymic squamous cell carcinoma.

## Background

Data from the Japanese Committee of Thoracic Surgery indicates that in 2020, mediastinal tumor resections comprised 5573 procedures in Japan. Among these, 2226, 341, and 354 procedures were due to thymoma, thymic carcinoma, and myasthenia gravis complicated with thymoma, respectively [1]. Notably, thymic carcinoma represented 6.1% of these cases. Thymic carcinomas are prone to invade surrounding structures, such as the major blood vessels (e.g., superior vena cava), pericardium, and pleura, usually necessitating extended surgical resections. However, due to its relative rarity, established treatment protocols for such cases remain elusive. Previous reports on thymic carcinomas suggest that complete resection and adjuvant therapy may be predictors of recurrence and prognosis [2–5]. Therefore, we present a case where surgical and perioperative management was performed under the presumption of thymoma with myasthenia gravis.

## Case presentation

**Fig. 1:**
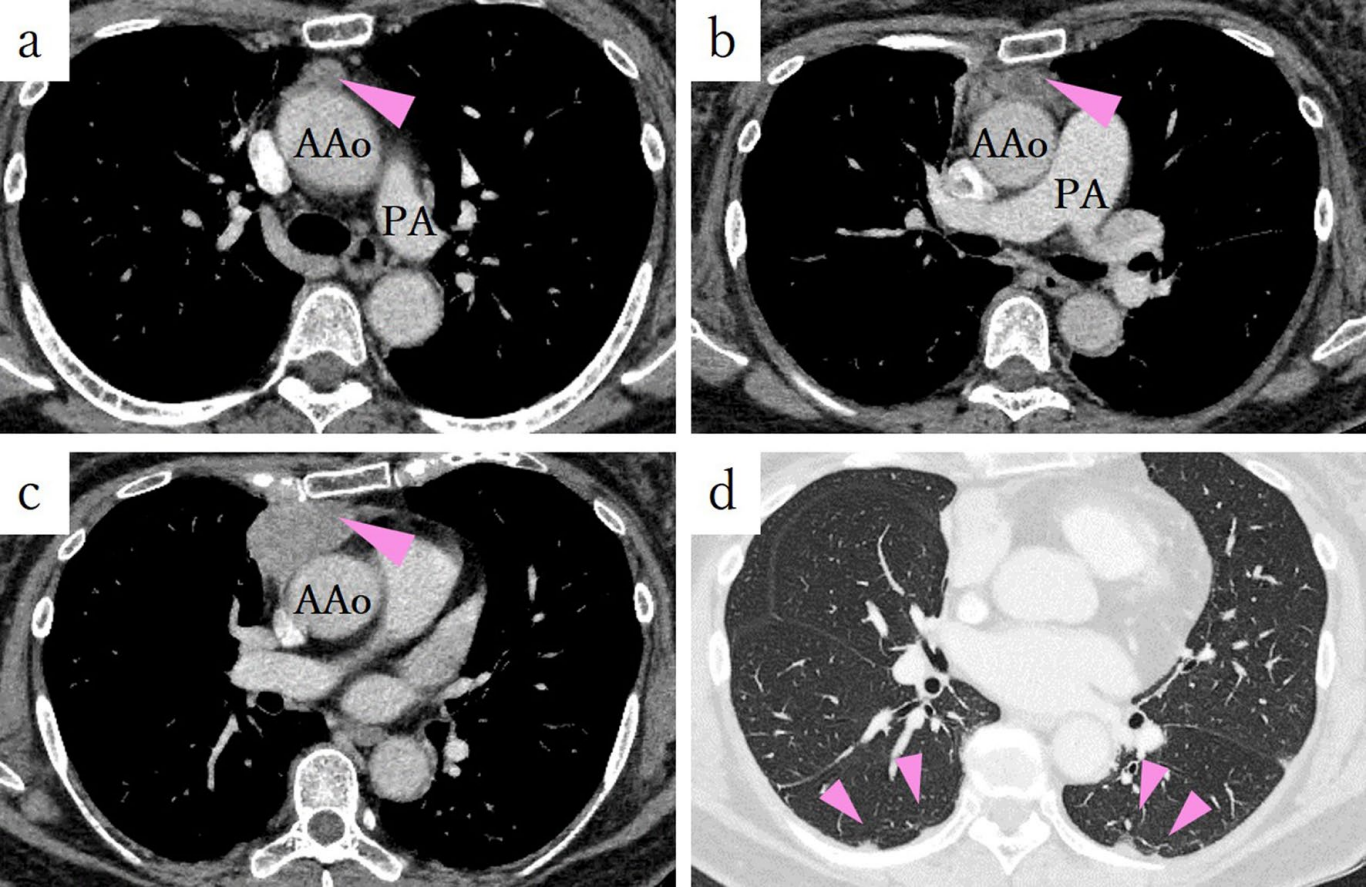
Preoperative CT shows tumors in the anterior mediastinum, measuring 15 × 14 × 10, 25 × 12 × 12, and 43 × 33 × 24 mm from the head (**a**, **b**, **c**). The two lower areas appeared to be continuous. **b**, **c** These findings suggested multicentric thymoma or intra-thymic metastasis. Furthermore, based on the tumor location, pericardial invasion, pleural dissemination (**d**), and a possible left adrenal adenoma were suspected. *AAo* ascending aorta, *PA* pulmonary artery, *CT* computed tomography

A 65-year-old female was referred to our department for a possible anti-acetylcholine antibody-positive thymoma complicated with myasthenia gravis. At the initial visit to the neurologist, she was diagnosed with myasthenia gravis of the ocular muscle type alone and was started on a cholinesterase inhibitor. Since the patient’s symptoms improved with this treatment, she was not treated with steroids preoperatively. A preoperative blood draw showed an elevated anti-acetylcholine receptor antibody titer of 4.7 nmol/L. Squamous cell carcinoma-related antigen was within the normal range at 0.9 ng/mL. Furthermore, preoperative CT revealed three distinct masses in the anterior mediastinum, measuring 15 × 14 × 10, 25 × 12 × 12, and 43 × 33 × 24 mm from the head (Fig. 1a, b, c). The two lower areas appeared to be continuous (Fig. 1b, c). These findings suggested multicentric thymoma or intra-thymic metastasis. Furthermore, based on the tumor location, pericardial invasion, pleural dissemination (Fig. 1d), and a possible left adrenal adenoma were suspected.

**Fig. 2:**
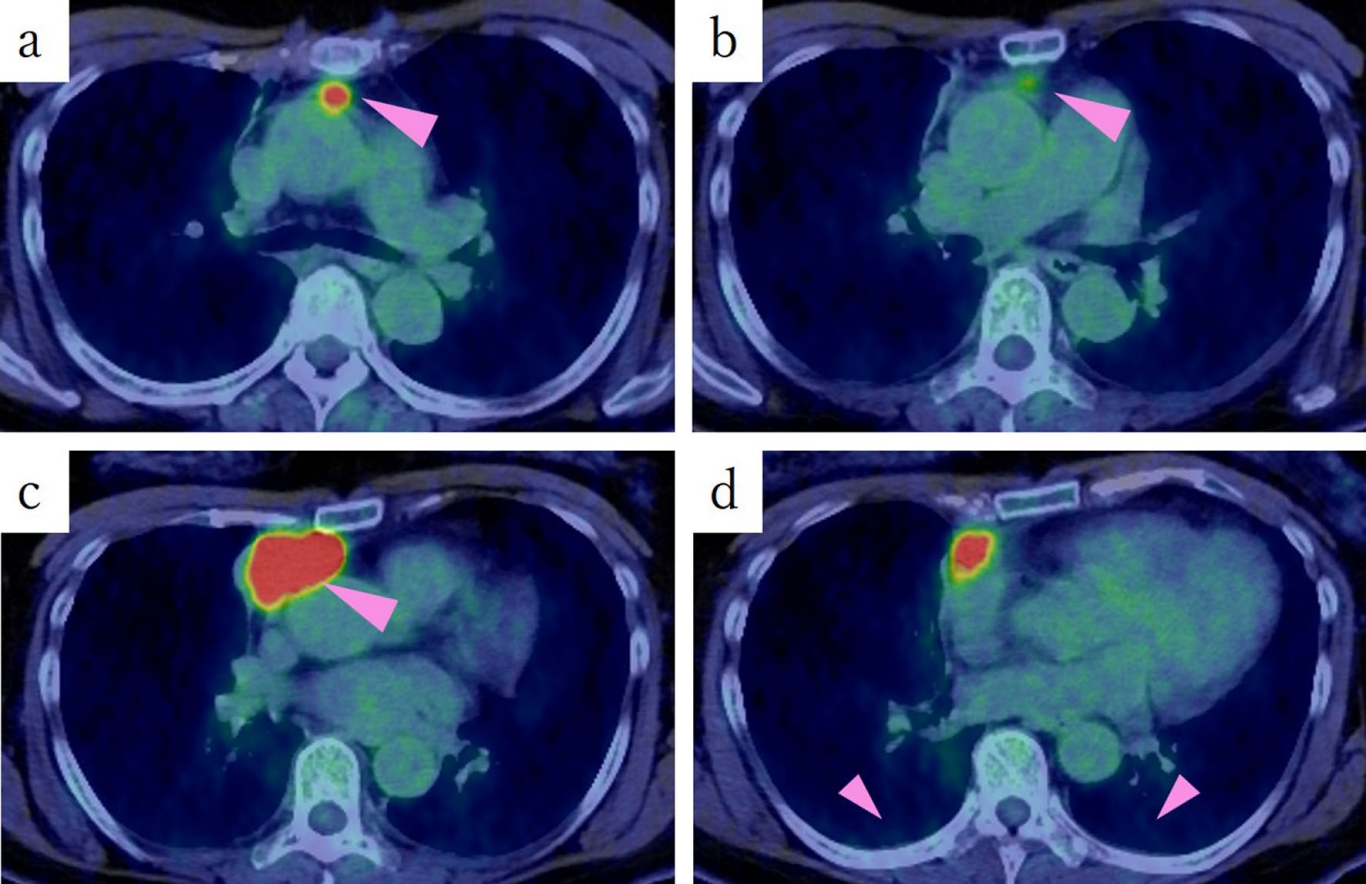
PET-CT shows an increased fluorodeoxyglucose uptake (SUV-max 8.5 and 18.5) in the anterior mediastinal tumor (**a**, **c**). No accumulation was observed in part of the tumor (**b**), the pleura (**d**), other lymph nodes or other organs. *PET-CT* positron emission tomography-computed tomography

To evaluate for suspected metastatic disease, a positron emission tomography-CT (PET-CT) was obtained. This revealed increased fluorodeoxyglucose uptake (SUV-max 8.5 and 18.5) in the anterior mediastinal tumor (Fig. 2a, c); however, no accumulation was observed in part of the tumor (Fig. 2b), the pleura (Fig. 2d), other lymph nodes, or other organs.

When she was admitted to the neurology department before the surgery, she complained of easy fatigue, and it was determined that a transition to generalized MG could not be ruled out.

After discussion, it was decided that steroids would be started postoperatively and intravenous immunoglobulin therapy would be administered due to the patient's coexisting diabetes and the fact that it was immediately before the surgery.

Following 5 days of preoperative intravenous immunoglobulin therapy to control her myasthenia gravis, in addition to anticholinesterase medication from the initial visit, the patient underwent surgery. The surgical procedure consisted of an extended thymectomy, pericardial resection, and reconstruction. Intraoperatively, no disseminated lesions were identified.

Definitive pathological examination revealed the co-occurrence of B3 thymoma and thymic squamous cell carcinoma. Moreover, the site of what appeared to be intra-thymic metastasis was metastasis to the anterior mediastinal lymph nodes (Figs. 1a, 2a). Therefore, lymph node metastasis was confirmed, classifying her condition as stage IV according to the UICC TNM Classification of Malignant Tumors (pT2N1M0, Stage IVa) and Masaoka staging system (Stage IVb).

Although the patient was successfully extubated postoperatively, she was admitted to the intensive care unit due to a rapid decline in her respiratory condition, which was suggestive of a myasthenic crisis. Difficult weaning from the ventilator necessitated a tracheotomy on the postoperative day 8. After the postoperative crisis onset, the patient was treated with anticholinesterase, tacrolimus, and 10 mg of prednisone via nasogastric tube starting in the first postoperative week. Concurrently, immunoabsorption plasmapheresis was administered seven times during the first 2 weeks postoperatively. On postoperative day 27, the patient received steroid pulse therapy; her respiratory condition gradually improved with ongoing supportive care, including blood purification therapy. The tracheal cannula was successfully removed on postoperative day 36.

Once she was medically stable, adjuvant radiotherapy at a dose of 60 Gy was initiated on postoperative day 34. She was discharged home on her last postoperative day and is ongoing outpatient follow-ups with serial imaging. At approximately 1 year postoperatively, no evidence of disease recurrence has been observed.

## Results

**Fig. 3:**
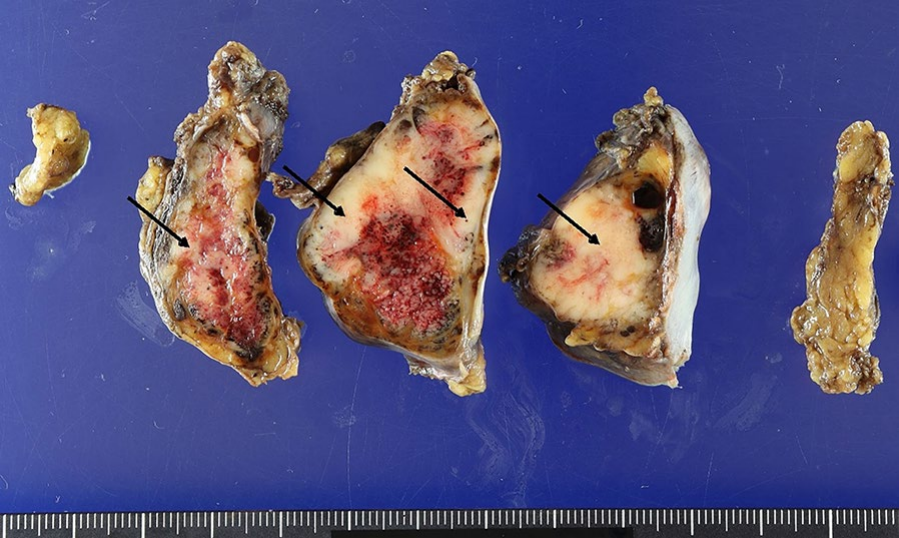
In the macroscopic findings, the white areas (black arrows) were mainly thymic squamous cell carcinoma

**Fig. 4:**
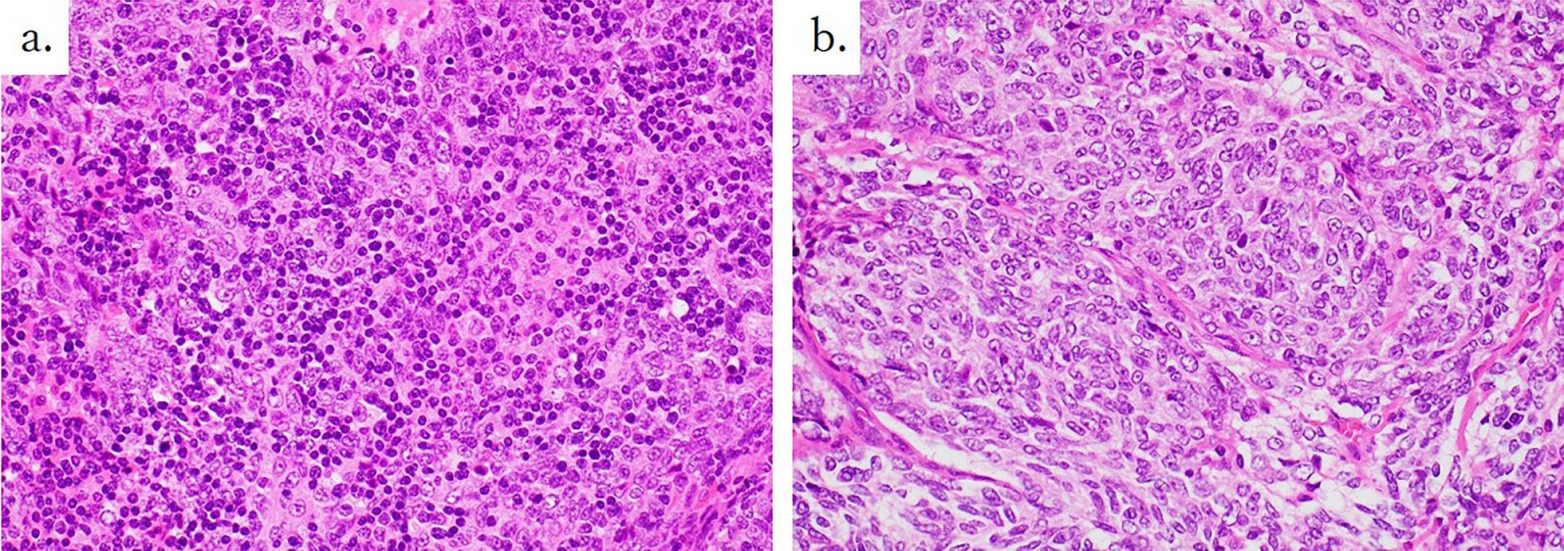
The final pathological results demonstrate the co-occurrence of B3 thymoma (**a**) and thymic carcinoma (**b**) on H&E staining. *H&E* hematoxylin & eosin

**Fig. 5:**
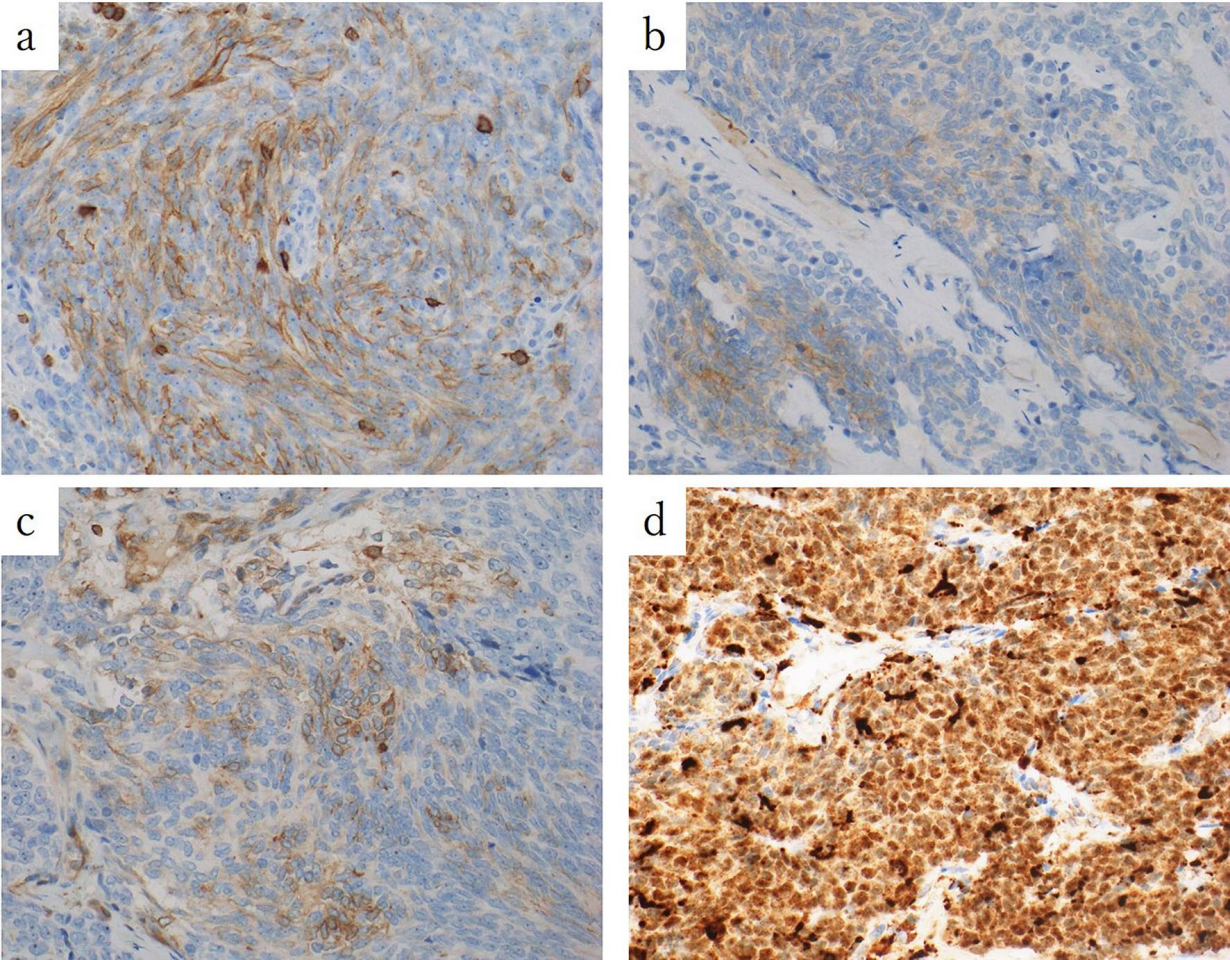
Immunohistochemical staining result showed that CD5 (**a**), c-kit (**b**), and Bcl-2 (**c**) were all weakly positive, and PAX8 was positive (**d**), confirming the diagnosis of thymic squamous cell carcinoma

Macroscopic findings revealed that the white areas were mainly composed of thymic squamous cell carcinoma (Fig. 3). The ratio of thymoma to thymic carcinoma was 1:1. In this case, based on the results of hematoxylin & eosin and immunohistochemical staining (Figs. 4, 5), the tumor was diagnosed as a mixture of B3 thymoma and thymic squamous cell carcinoma. Upon detailed examination, the tumor on the right side of the thymus was classified as a mixed tumor, with a mixture of thymoma and thymic carcinoma components within a single tumor lesion. Generally, the tumors displayed a “thymic carcinoma in thymoma” pattern, with thymic carcinoma areas within thymomas, and the histomorphology suggested thymic squamous carcinoma arising from thymoma. Ultimately, the anterior mediastinal lymph node metastasis was determined to be of thymic squamous cell carcinoma origin.

When comparing the images, among the three tumors observed on CT, the most cephalic tumor had metastasis to the anterior mediastinal lymph node; the caudal tumor, which was undetected on PET-CT, was identified as thymic tissue; and the most caudal tumor was a mixed tumor of thymoma and thymic carcinoma.

## Discussion

Combined thymic carcinoma, a rare condition comprising thymoma and thymic carcinoma, arises primarily within the thymus with a reported incidence of < 1% [6]. For the thymoma component, these tumors predominantly harbor a B2 or B3 thymoma, whereas types A and AB are less frequently encountered [6]. The most commonly reported combinations involve squamous cell carcinoma with B3 thymoma, A thymoma, papillary adenocarcinoma, or sarcomatoid carcinoma [7].

To the best of our knowledge, no reports of thymomas complicated with carcinoma and myasthenia gravis exist. In this case, the presence of squamous cell carcinoma suggests the possibility of a combined thymic carcinoma, with the malignant component and myasthenia gravis likely attributable to the B3 thymoma.

Furthermore, what appeared as a multicentric thymoma on preoperative imaging was ultimately proven to be metastasis to the anterior mediastinal lymph node. Previous reports have shown that lymph node metastasis occurs in 1.8% and 26.8% of thymomas and thymic carcinomas, respectively [8]. Therefore, the identification of a hypermetabolic lymph node on PET-CT, particularly in the setting of equivocal findings for lymph node metastasis, should raise suspicion for coexistent thymic carcinoma.

While the concept of combined thymic carcinoma has been removed from the fifth edition of the World Health Organization classification, thoracic surgeons should remain aware of this potential clinical condition.

## Conclusion

The potential for myasthenia gravis to complicate thymomas, particularly B3 thymomas, which can present as combined thymic squamous cell carcinoma, warrants consideration during preoperative imaging evaluation and should be explained during patient education.

## Data Availability

The patient data of this case report will not be shared to ensure patient confidentiality.

## Abbreviations

CT: Computed tomography
UICC: The Union for International Cancer Control
PET-CT: A positron emission tomography-CT
